# Comparative Geno-Plasticity Analysis of *Mycoplasma bovis* HB0801 (Chinese Isolate)

**DOI:** 10.1371/journal.pone.0038239

**Published:** 2012-05-31

**Authors:** Jingjing Qi, Aizhen Guo, Peng Cui, Yingyu Chen, Riaz Mustafa, Xiaoliang Ba, Changmin Hu, Zhidi Bai, Xi Chen, Lei Shi, Huanchun Chen

**Affiliations:** 1 National Key Laboratory of Agricultural Microbiology, Huazhong Agricultural University, Wuhan, China; 2 College of Veterinary Medicine, Huazhong Agricultural University, Wuhan, China; The University of Hong Kong, China

## Abstract

*Mycoplasma bovis* pneumonia in cattle has been epidemic in China since 2008. To investigate *M. bovis* pathogenesis, we completed genome sequencing of strain HB0801 isolated from a lesioned bovine lung from Hubei, China. The genomic plasticity was determined by comparing HB0801 with *M. bovis* strain ATCC® 25523™/PG45 from cow mastitis milk, Chinese strain Hubei-1 from lesioned lung tissue, and 16 other *Mycoplasmas* species. Compared to PG45, the genome size of HB0801 was reduced by 11.7 kb. Furthermore, a large chromosome inversion (580 kb) was confirmed in all Chinese isolates including HB0801, HB1007, a strain from cow mastitis milk, and Hubei-1. In addition, the variable surface lipoproteins (*vsp*) gene cluster existed in HB0801, but contained less than half of the genes, and had poor identity to that in PG45, but they had conserved structures. Further inter-strain comparisons revealed other mechanisms of gene acquisition and loss in HB0801 that primarily involved insertion sequence (IS) elements, integrative conjugative element, restriction and modification systems, and some lipoproteins and transmembrane proteins. Subsequently, PG45 and HB0801 virulence in cattle was compared. Results indicated that both strains were pathogenic to cattle. The scores of gross pathological assessment for the control group, and the PG45- and HB0801-infected groups were 3, 13 and 9, respectively. Meanwhile the scores of lung lesion for these three groups were 36, 70, and 69, respectively. In addition, immunohistochemistry detection demonstrated that both strains were similarly distributed in lungs and lymph nodes. Although PG45 showed slightly higher virulence in calves than HB0801, there was no statistical difference between the strains (*P*>0.05). Compared to Hubei-1, a total of 122 SNP loci were disclosed in HB0801. In conclusion, although genomic plasticity was thought to be an evolutionary advantage, it did not apparently affect virulence of *M. bovis* strains in cattle.

## Introduction

*Mycoplasma bovis* is a member of the *Mycoplasmataceae* family in the class of Mollicutes that was first identified as a causative agent of mastitis in 1961 and recognized as an important pathogen of bovine respiratory disease in 1976 [1]. *M. bovis* pneumonia became more common with the development of the beef industry and was often induced by long-distance transport of stockers to feedlots. Because this microorganism is resistant to several antibiotics including β-lactams and there is no effective commercial vaccine available, *M. bovis* has caused a significant economic loss in the United States, Canada and most of Europe [2], [3]. In China, *M. bovis* pneumonia was first reported in 2008 in the Hubei province with an average case fatality of 10%, but possibly over 40% [4].

Although *M. bovis* was discovered nearly five decades ago, its pathogenic mechanisms remain largely unknown. Recently, the complete genomic sequences of *M. bovis* PG45 [5] and a Chinese strain Hubei-1 [6] have been published and the genomic annotation has identified some putative virulent genes, which are yet to be confirmed. To obtain more insight into *M. bovis*, we sequenced another Chinese strain, HB0801, isolated from Hubei province in 2008. In addition, by assembling the full genomic sequences, we preformed comparative genomic analysis of *M. bovis* strains HB0801, PG45, Hubei-1 and 16 other sequenced *Mycoplasmas*. Our results revealed that HB0801 contained extraordinary genomic plasticity. Further, in vivo cattle experiments demonstrated that both PG45 and HB0801 had similar virulence to calves.

## Materials and Methods

### Strain and culture

The HB0801 strain was isolated from the lesioned lung of an infected beef cattle from Yingcheng city in Hubei province, China by this laboratory [4] and stored at the China Center for Type Culture Collection (CCTCC # M2010040) at Wuhan University, Wuhan, China. HB1007 was isolated from milk of a dairy cow with mastitis in Hubei in 2010.

The strain was cultured on a pleuropneumonia-like organisms (PPLO) agar plate at 37°C, in a 5% CO_2_ atmosphere for 3 days or in PPLO broth (2.5 g glucose, 10.5 g PPLO, 2.5 g yeast, 50 mL donor equine serum (Thermo Fisher Scientific, Waltham, MA, USA), 5 mL10% arginine, 5 mL 10×MEM, 5 mL of 80,000 IU/mL penicillin-G, and 500 µL 1% phenol red) at 37°C for 3 days on an orbital shaker.

### Library construction and DNA sequencing

The culture was harvested from 50 mL broth by centrifugation at 1180 g for 30 min and DNA was extracted using a bacterial genomic DNA extraction kit (Tiangen, Beijing, China) and sent to the Tianjin Biochip Corporation (Tianjin, China) for further processing.

A 6 kb library of the HB0801 genome was prepared by standard protocols at the Tianjin Biochip Corporation and sequenced with a Roche 454 GS-FLX Pyrosequencer (Roche, Welwyn Garden City, Hertfordshire, UK) according to the manufacturer's protocols. The 284,110 paired reads with an average length of 136 bps, as well as 52,910 single end reads with an average length of 324 bps were produced, representing a 56.6-fold coverage of the genome. 93.6% (315,507) of reads were assembled *de novo* using the 454/Roche Newbler assembly program (v2.0). The assembly produced 8 scaffolds and 134 non-redundant contigs in total. The N50 contig length of 76 large contigs (>1 kb) was 30,795 bp and the largest one was 78,194 bp. The number of total bases of the large contigs was 908,485 bp.

To fill the gaps within the scaffolds and validate the sequences from the assembly, an additional 2 kb library was prepared using Illumina sample preparation kits and sequenced by using an Illumina Solexa GA IIx (Illumina, Little Chesterford, Essex, UK) according to the manufacturers' guidelines. A total of 6,278,608 reads with 54 bp lengths were generated to reach a 342.8-fold coverage.

After removal of duplications, all generated reads were mapped to the scaffolds constructed by 454 reads to yield an assembly using the Burrows-Wheeler Alignment tool (BWA) [7]. The gaps within the scaffolds were filled using Solexa sequencing technology (Illumina, Inc., San Diego, CA, USA) and 454 paired-end reads with one end mapped on the unique contig and the other end located in the gap region. The local assembly was performed using an in-house Perl script. In addition, the combination of the Solexa and 454 sequencing helped to solve the possible errors of small indels in homopolymers [8].

### Genome annotation and analysis

When the genomic sequencing was completed, no *M. bovis* genomic sequences had been published or available for reference. The HB0801 open reading frames (ORFs) were initially predicted using Glimmer 3 software (http://www.cbcb.umd.edu/software/glimmer/) and most were verified using the tBLASTn algorithm (http://blast.ncbi.nlm.nih.gov/) and compared to the related *M. agalactiae* genome (GenBank Accession: NC_009497.1). Transfer RNA (tRNA) and ribosomal RNA (rRNA) genes were predicted using the tRNAscan-SE program (http://lowelab.ucsc.edu/tRNAscan-SE/) and by similarity to *M. agalactiae* rRNA genes. The Artemis algorithm [9] was used to collate data and facilitate annotation. Functional predictions were based on BLASTp algorithm (http://blast.ncbi.nlm.nih.gov/Blast.cgi?PAGE=Proteins) similarity searches against the UniprotKB database (http://www.ebi.ac.uk/uniprot) and the clusters of orthologous groups (COG) database (http://www.ncbi.nih.gov/COG). Lipoproteins (LPs) were determined using *preg* in the EMBOSS package [10]. The PROSITE expression of the extended lipobox search pattern was obtained from previous work on strain PG45 [5] and translated into regular expression. In addition, signal peptide sequences and putative transmembrane proteins were predicted using SIGNALP [11], and TMHMM 2.0 [12], respectively. Furthermore, the inter-strain comparative analysis for *M. bovis* strains was performed using Mauve 2.3.1 genome alignment software [13] and the Artemis Comparison Tool (ACT) [14].

### Orthologs detection and phylogenetic analysis

The genomes of 17 *Mycoplasmas* strains were freely available at the time of the study and were presented in Table 1. Coding sequences (CDS) were extracted from GenBank files, and orthologs or recent paralogs were determined using OrthoMCL [15]. This program first made a tBLASTn search, which helped to detect frame shifts and truncated genes, and predict the putative pseudogenes and missed genes in annotation. Then we performed reciprocal BLASTP searches of the 17 proteomes to define the ortholog pairs based on the clustering criteria; 10^−10^ cut-off e-value, minimum protein length of 40 amino acids and at least 70% identity. Putative orthologs or paralogs were clustered into protein families using the Markov Cluster algorithm (MCL) [16] with an inflation parameter value of 1.5. In return, the MCL yielded a set of clusters, with each containing a set of orthologs and/or recent paralogs. We used the OrthoMCL output to construct a table describing the gene content of various *Mycoplasma* genomes (Table S1). Genes that were not included in a cluster were considered taxon-specific genes. This table was used to construct core-genome data sets of *Mycoplasma* (Table S2).

**Table 1:** Genomes Used for Phylogenetic Construction and Comparison Analysis.

Species	Accession No. in GenBank
*Mycoplasma agalactiae* PG2*	NC009497
*Mycoplasma agalactiae* 5632	NC013948
*Mycoplasma arthritidis* 158L3-1*	CP001047
*Mycoplasma capricolum subsp. capricolum* ATCC 27343*	CP000123
*Mycoplasma conjunctivae* HRC/581*	FM864216
*Mycoplasma gallisepticum* str. R(low)*	AE015450
*Mycoplasma hominis* ATCC 23114*	FP236530
*Mycoplasma hyopneumoniae* 232*	AE017332
*Mycoplasma hyopneumoniae* 7448	AE017244
*Mycoplasma hyopneumoniae* J	AE017243
*Mycoplasma mobile* 163K*	AE017308
*Mycoplasma mycoides subsp. capri* str. GM12	CP001621
*Mycoplasma mycoides subsp. mycoides* SC str. PG1*	BX293980
*Mycoplasma pneumoniae* M129*	U00089
*Mycoplasma pulmonis* UAB CTIP*	AL445566
*Mycoplasma synoviae* 53*	AE017245
*Mycoplasma bovis* HB0801*	CP002058

Note:

* , *Mycoplasma* species involved in the frequency of orthologs analysis.

The orthologs did not need to be conserved in all codons. Frame shifts [17], gene mergers or sequencing errors [18] could greatly interrupt the amino acid sequences of pseudogenes. Orthologs were first compared at the amino acid level with BLASTp to retrieve all conserved regions. The codons in the non-conserved regions were removed using the in-house Perl scripts and edited manually. The remaining amino acid sequences were aligned using the Clustal W 1.82 algorithm [19] and then back-translated to DNA using an in-house Perl script.

The phylogenetic tree of each ortholog was re-constructed from the DNA alignment with the phyML algorithm (http://www.atgc-montpellier.fr/phyml/) using the maximum likelihood under the GTR+ gamma (with 8 categories) +I model of evolution with a BioNJ start tree [20]. The 1000 bootstrap experiments were performed on the concatenated sequences to assess the topological robustness. The phylogenetic tree of each ortholog was resolved to several independent bipartitions, each of which represented one branch of the phylogenetic tree. Support for each bipartition was obtained by bootstrapping a maximum likelihood tree search using Tree-puzzle 5.2 [21]. All well-supported (>70% bootstrap support) bipartitions from each ortholog were collected. The super-tree was re-constructed using the matrix representation with parsimony (MRP) method [22] as implemented in Clann 2.0.2 [23].

### Horizontal gene transfer (HGT) analysis

As a gene was being moved laterally, the depicted phylogenetic relationship would be different from the typical species tree [24]. If one *M. bovis* ortholog was clustered with other species in bipartitions at a >70% bootstrap support level, but not with *M. agalactiae*, this ortholog might have undergone a putative recombination. This method was utilized to detect recombination between HB0801 and other *Mycoplasmas* species with help of the phylogenetic tree of each ortholog and species trees obtained beforehand.

### Sequence confirmation with PCR

In order to confirm *vsp* gene sequences, we designed a pair of primers for the flanking sequences of the entire *vsp* cluster region.

Vsp-F: 5′-TGCTATTCATTTCTTTGTAGTATTTTATGT-3′;

Vsp-R: 5′-TTTATTTCCTTTACCAATTACATATATTCG-3′.

PCR assays were conducted using 2 µL HB0801 genomic DNA as the template in 25 µL reaction mixture with 1 U of LA Taq DNA polymerase (TaKaRa, Tokyo, Japan) in 1×buffer supplied by the manufacturer, 200 µM dNTPs and 1.4 µM of each primer. The amplification was programmed over 35 cycles, each consisting of 45 s at 95°C, 30 s at 60°C, and 8 min at 72°C and an initial denaturation step at 95°C for 5 min.

In order to show the inverted region of the HB0801 genome as compared to *M. bovis* PG45, we designed two other sets of primers: Inv-1 and Inv-2, which were specific to the two connection regions at both ends of the inversion.

Inv-1F: 5′-GAAAAATGGAACTCCTTTACCTTATGG-3′;

Inv-1R: 5′-AATGAGATAGAACTGTTAGGAGGCGTC-3′.

Inv-2F: 5′-GCATCACCATTTGTTCTGTTGTCTGTT-3′;

Inv-2R: 5′-CTGACGCTGCTGCTTATGATTTTATTG-3′.

Both PCR reactions were programmed in the cycler (Veriti 96-Well Thermal Cycler, Applied Biosystems) for 30 cycles, each consisting of 40 s at 94°C, 40 s at 53°C, and 90 s at 72°C and an initial denaturation step of 94°C for 5 min. These reactions were expected to yield the 2307 bp and 2893 bp fragments, respectively, if the inversion occurred. The products were sequenced at Sangon Biotech (Shanghai) Co. Ltd., Shanghai, China.

### Pathological assessment of *M. bovis* strains in cattle

To compare the virulence of *M. bovis* strains HB0801 and PG45, cattle were artificially infected. The animal treatment was carried out in strict accordance with the Hubei Regulations for the Administration of Affairs Concerning Experimental Animals, 2005. The protocol was approved by the China Hubei Province Science and Technology Department (Permit Number: SYXK(ER) 2010-0029). A total of nine locally bred calves (age: ∼6 months) without overt clinical signs were purchased from a local market and raised at the Huazhong Agricultural University experimental farm. The calves were randomly divided into three groups (n = 3 calves each): two infection groups and a control group. In the infection groups, calves were challenged through intratracheal injection with 10 mL (10^9^ CFU/mL) of *M. bovis* strains HB0801 or PG45 for three successive days, while the remaining calves were mock-infected with an equal amount of medium as a negative control. After challenge, each group was segregated in different pens and observed for 20 days.

At day 20 post-challenge, all calves were euthanatized by intravenous injection of sodium pentabarbitone and postmortem examinations were performed. The scoring system for the gross pathological lesions of the inner organs [25] and lungs [26] were applied to assess virulence of the strains. The lung tissue and cervical lymph nodes (1 cm^3^) were cut and fixed immediately with 10% neutral buffered formalin and sent to Wuhan Guge Biological S & T Co. Ltd. (Wuhan, China) to produce 4 µm-thick paraffin-embedded tissue sections. Immunohistochemical staining by the streptavidin–biotincomplex (SABC) method and SABC (mouse IgG) - POD kit was performed according to the manufacturer's instructions (Boster Biotech, Wuhan, China). Each tissue from one calf was sub-divided into 2 groups of 3 sections each: (1) Negative control: the sections were stained with phosphate-buffered saline (PBS) instead of mouse monoclonal antibody to *M. bovis*; (2) *M. bovis* staining: the sections were stained with in-house mouse monoclonal antibody 2A3 (1∶200 dilution) to *M. bovis* [27]. All sections were stained with biotinylated goat anti-mouse IgG and then Streptavidin–HRP complex. The reaction was developed with 3,3′-Diaminobenobenzidine (DAB)/H_2_O_2_, counter-stained with hematoxylin and mounted. Results were positive when the cells stained brown and negative when the cells stained blue. Based on cell density and uniformity, lymph nodes were selected to quantitatively analyze the difference between positive signals of infection and control groups with Image-Pro Plus 6.0 (IPP6) software (Media Cybernetics, Inc., Bethesda, MD, USA). Briefly, six fields for each tissue were photographed under a light microscope (×400) and positive signals for each image was assessed by IPP6 as the integrated optical density (IOD), and the total IOD of each calf was calculated. The difference in IOD between each group was analyzed with R*64 2.13.0 software. Differences were significant when *P*<0.05 and very significant when *P*<0.01.

### Nucleotide Sequence Accession Number

The complete HB0801 genome sequence was deposited in the GenBank database (Accession number: CP002058).

## Results

### General features

**Figure 1:**
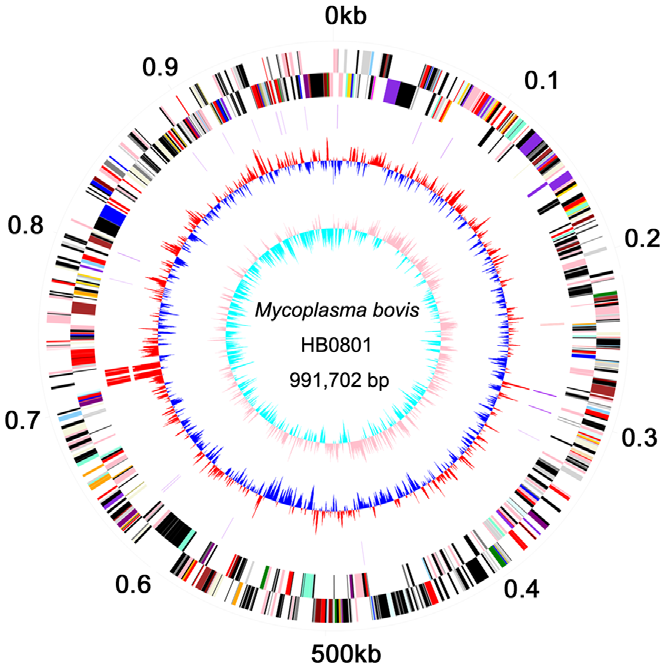
Circular Diagram of the *M. bovis* HB0801 Genome Structure. The *dna*A gene is at position zero. Starting from the outside, the first circle shows the genome length (units in Mb); the second and the third circles show the locations of the predicted CDSs on the plus and minus strands, respectively, which were color-coded by COG categories (gold for translation, ribosomal structure and biogenesis; orange for RNA processing and modification; light orange for transcription; dark orange for DNA replication, recombination and repair; antique white for cell division and chromosome partitioning; pink for defense mechanisms; red for signal transduction mechanisms; peach for cell envelope biogenesis and outer membrane; deep pink for intracellular trafficking, secretion and vesicular transport; pale green for post-translational modification, protein turnover and chaperones; royal blue for energy production and conversion; blue for carbohydrate transport and metabolism; dodger blue for amino acid transport and metabolism; sky blue for nucleotide transport and metabolism; light blue for coenzyme metabolism; cyan for lipid metabolism; medium purple for inorganic ion transport and metabolism; aquamarine for secondary metabolites biosynthesis, transport and catabolism; and gray for unknown function); the fourth circle shows tRNAs (violet) and rRNAs (red); the fifth circle shows the centered GC (G+C) content of each CDS (red: above mean and blue: below mean); and the sixth circle shows the GC (G+C) skew plot.

The genome of *Mycoplasma bovis* HB0801 was composed of 991,702 base pairs (bps) with a single circular chromosome (Figure 1). The general genomic features of *M. bovis* HB0801 were compared with strains Hubei-1 and PG45 (Table 2). The genome size of HB0801 was about 11.7 kb smaller than that of PG45. This difference comprises 1% of the PG45 genome. The common properties between HB0801 and PG45 include 29.3% of G+C content, 34% of tRNAs, a similar coding percentage (84.2% for HB0801, 83% for PG45), similar length of CDSs (1096 bps for HB0801, 1089 bps for PG45), and similar number of insertion sequences (IS). However, there was a large difference in the number of integrative conjugative elements (ICE) and variable surface lipoproteins (*vsp*).

**Table 2:** General Feature Comparison of *Mycoplasma bovis* HB0801 with Hubei-1 and *Mycoplasma bovis* ATCC® 25523™/PG45.

Features	Mycoplasma bovis strains		
	HB0801	Hubei-1^a^	PG45^b^
Genome size (bp)	991,702	948,121	1,003,404
G+C content (%)	29.3	29.4	29.3
Protein coding genes without pseudogenes	762	803	765
Pseudogenes	46	30	61
Average length of CDSs (bp)	1097	1058	1089
Percentage coding (%)	84.2	89.5	83
tRNA	34	34	34
rRNA (23S, 16S and 5S) sets	2	1	2
Predicted lipoproteins	103^c^	96^c^	96
Insertion sequences	51	26	54
ICE Number	1	1	2
vsp cluster genes	6	0	13

a Li et al., 2001;

b Wise et al., 2010. ICE, Integrative Conjugative Element; *vsp*, Variable Surface Lipoproteins.

c The data were predicted using the same method mentioned in PG45 genome paper.

### Insertion sequence (IS) elements

DNA sequence analysis and BLASTp searches against the IS database (http://www-is.biotoul.fr/is.html) found 51 IS elements in the HB0801 genome that belonged to eight distinct categories. These categories of IS elements were designated as IS*Mbov* (1–8)_HB0801_ and each had a different copy number, ranging from 1 to 12. The features of these 8 categories of IS elements including the amine acid sizes of ORF, copies in *M. bovis* HB0801 strain, homology to other IS elements and the origins and the related IS families were shown in Table 3. The first seven categories (IS*Mbov*1-7_HB0801_) were identical to IS*Mbov*1-7_PG45_ of PG45, as previously described [28]. IS*Mbov*8_HB0801_ was only found in HB0801 and showed 26% similarity to either IS*Mmy*1 in *M. mycoides subsp. mycoides small colony type* or IS*Mbov*2 in PG45.

**Table 3:** Features of IS Elements in *Mycoplasma bovis* HB0801.

IS	Copies	Amino acid size	Homology to other IS elements	Origin	IS family
IS*Mbov*1_HB0801_	12(2 p)	416	94%ISMag1	*M. agalactiae* PG2	IS30
IS*Mbov*2_HB0801_	6(3 p)	470	94%IS*Mmy*1	*M. mycoides* SC PG1	IS1634
IS*Mbov*3_HB0801_	9(1 p)	557	94%IS1634	*M. mycoides* SC PG1	IS1634
IS*Mbov*4_HB0801_	4(2 p)	477	34%ISMmy1	*M. mycoides* SC PG1	IS1634
IS*Mbov*5_HB0801_	6(2 p)	462	28%ISMmy1	*M. mycoides* SC PG1	IS1634
IS*Mbov*6_HB0801_	3	338	39%IS1630	*M. fermentans* PG18	IS30
IS*Mbov*7_HB0801_	10(3 p)	414	42%ISMmy2	*M. mycoides* LC	IS3
IS*Mbov*8_HB0801_	1	478	26%ISMmy1	*M. mycoides* SC PG1	IS1634

Notes: p, Pseudogene which is interrupted or truncated, containing premature stop codon or frameshift mutation.

### Integrative conjugative element (ICE)

The integrative conjugative element B-1 of *M. bovis* HB0801 designated as ICEB_HB0801_-1 (*Mbov*_0384-0400) was found, but ICEB-2 was absent in the HB0801 genome as compared to PG45. The BLASTp comparative analysis between ICEB_HB0801_-1 and ICEs of other *Mycoplasmas* indicated that ICEB_HB0801_-1 was best aligned with ICEA_PG2_ of *M. agalactiae* PG2, suggesting that they occurred in a common ancestor prior to speciation. Between ICEB_HB0801_-1 and ICEB_PG45_-1, almost all the corresponding CDSs showed an average of 99% identities, except CDS11 and CDS5, which were designated according to previous *M. fermentans* ICEs nomenclature [29]. The CDS11 (*Mbov*_0392) presented one copy in ICEB_HB0801_-1, two copies in ICEB_PG45_-1 and both shared an average of 55% identities. The CDS5 (*Mbov*_0387), which encoded TraG, was disrupted by a stop mutation in ICEB_HB0801_-1. This was a conjugal protein that coupled the relaxosome to the translocation apparatus [30]. There was also another conjugation-related protein, TraE (*Mbov*_0397) in *M. bovis* HB0801, which was thought to be involved in DNA transport across the conjugative pore [31], [32].

*M. bovis* PG45 ICEB-2 also contained the *traG* gene and the *traE*-like ICEB-1pseudogene. Interestingly, there were low identities of these two genes between ICEB-1 and ICEB-2 in PG45. The other ICEB-2 genes encoded transposases, putative lipoproteins, membrane proteins and hypothetical proteins without known functions. Furthermore, all showed no identities to those of ICEB-1. Therefore, the loss of ICEB-2 in HB0801 may be related to a strain level difference and host-pathogen interactions.

### Variable surface lipoproteins (Vsp) cluster

**Figure 2:**
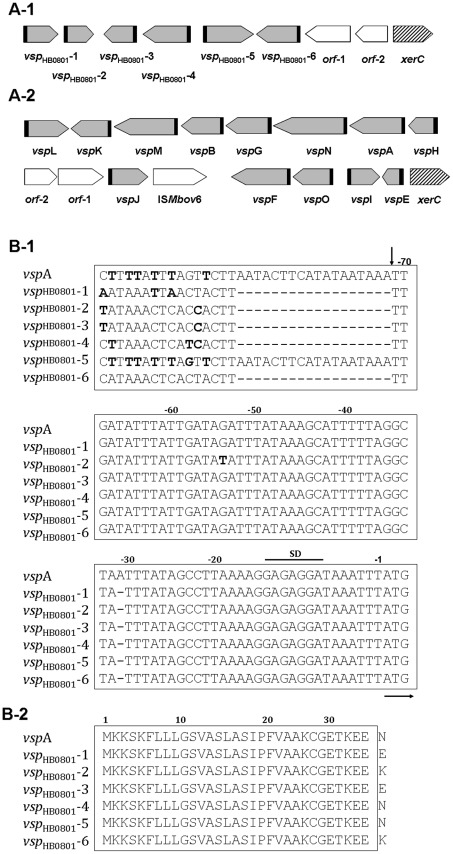
Comparison of *vsp* Gene Cluster between *M. bovis* HB0801 and PG45. The *vsp* gene loci of HB0801 (A-1) and PG45 (A-2) are shown. The locations and directions of the *vsp* ORFs are indicated with gray arrows. The adjacent non-*vsp* ORFs (ORF-1 and ORF-2) are indicated with open arrows. The locations of the putative tyrosine recombinase genes (*xer* C) are indicated with hatched arrows. The highly homologous regions upstream of the *vsp* genes are indicated with black blocks. (B) Sequence alignments for *vsp* upstream 5′ regions (B-1) and Vsp N-terminal regions (B-2). The HB0801 *vsp* gene family (*vsp*_HB0801_-1 to -6) and the *vsp*A gene, a representative PG45 *vsp* gene, are compared. The numbers above the sequences indicate positions relative to the initiation codon. Nucleotides representing a putative ribosome-binding site (SD) are headlined and the initiation codon (ATG) is underlined with a horizontal arrow. The division of the *vsp* upstream 5′ region into two distinct cassettes is indicated by a vertical arrow at nucleotide position -72.

A cluster of 6 *vsp*-related ORFs (*Mbov*_0793-*Mbov*_0798) was found in HB0801 and designated as *vsp*_HB0801_-1 to 6, while PG45 had 13 *vsp*-related ORFs (Figure 2A). With the exception of *vsp*_HB0801_-6 (Mbov_0798), which had a 99% identity to PG45 *vsp*L, none of the *vsp*-related ORFs in HB0801 was identical to the PG45 *vsp* genes, which have been already characterized. Similar to the structure of *vsp*_PG45_ genes, each *vsp*_HB0801_ gene contained two highly conserved upstream regions and a variable C-terminal repetitive downstream region. The highly conserved regions contained a non-coding 70 bp sequence upstream from the ATG initiation codon (Figure 2B-1) and a fragment encoding 31 amino acids at the N-terminal region (Figure 2B-2), which represented a typical prokaryotic lipoprotein signal peptide. In addition, some non-*vsp* ORFs (ORF-1 and ORF-2, Figure 2A) in this cluster and the adjacent site-specific tyrosine recombinase were shown to be highly conserved in both strains.

### Comparison between HB0801 and PG45

**Figure 3:**
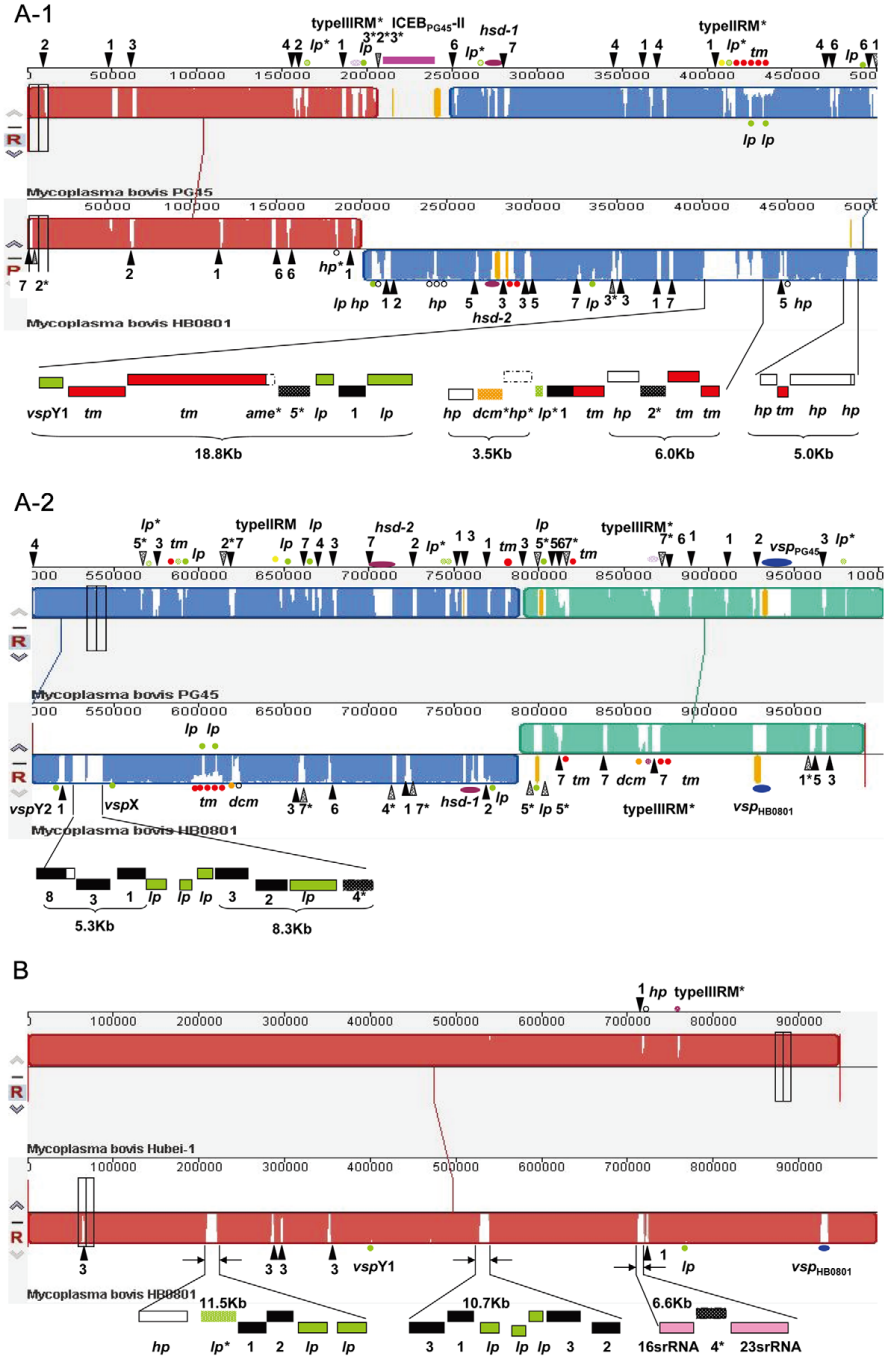
Genomic Comparison of *M. bovis* strains HB0801, PG45 and Hubei-1. (A) Complete genome comparison between HB0801 and PG45. A-1 and A-2 represent the upstream and downstream regions of the inversion breaking sites. (B) Genome comparison between HB0801 and Hubei-1. The middle blue region of HB0801 represents the large inverted fragment compared to PG45. The numbers 1–7 represent IS*Mbov*1-IS*Mbov*7, respectively (black triangles); lp: putative lipoprotein gene (green dots); tm: putative transmembrane protein gene (red dots); *hsd*: type I restriction modification system (purple dots); *dcm*: DNA-methyltransferase gene (orange dots); *vsp*: variable surface lipoprotein (blue dots); *hp*: hypothetical protein gene (hollow dots). The asterisks mark pseudo genes.

The genome of HB0801 was compared to PG45 using the Mauve 2.3.1 software (Figure 3A-1 and 3A-2). A large mid-inversion region of about 580 kb was discovered in the HB0801 compared to PG45, that also existed in Hubei-1 genomes [6]. The mechanism leading to this inversion was explored. The analysis revealed that two mobile genetic elements were associated with this inversion. A complete IS*Mbov*3 and an interrupted IS*Mbov*3 were separately found at each side of the inverted fragment in PG45 but were deleted on both sites of this inversion in HB0801. In addition, another mobile element ICEB-2 was upstream of the inversion region in PG45, but completely absent in HB0801 and Hubei-1 [6]. Therefore, these mobile genetic elements might have mediated this inversion mutation.

The difference between *M. bovis* strains HB0801 and PG45 was mainly due to IS elements. Generally speaking, ISs are distributed stochastically across the whole genome. With the exception of IS*Mbov*8, which was absent in PG45, both strains contained IS*Mbov*1 to IS*Mbov*7. However, the copy number and location of each IS element were different.

In addition to IS elements, there were some unique insertion fragments in HB0801 compared to PG45. One large insertion fragment (*Mbov*_0339-*Mbov*_0350) was 18.8 kb, other smaller insertions included fragments of 3.5 kb (*Mbov*_0354-*Mbov*_0357), 6.0 kb (*Mbov*_0365-*Mbov*_0368), 5.0 kb (*Mbov*_0417-*Mbov*_0420), 5.3 kb (*Mbov*_0454-*Mbov*_0457) and 8.3 kb (*Mbov*_0463-*Mbov*_0466), all which were shown in Figure 3A-1 and 3A-2. With the exception of the 5.0 kb fragment, each insertion contained at least one IS element in or outside the region and therefore the IS elements might be responsible for the gene transfer. In addition to the IS elements, most of the genes included in the insertion fragments were encoding putative lipoproteins or transmembrane proteins (marked with green or red color), those would probably result in the virulence and the phenotype differences between two strains.

*M. bovis* strains HB0801 and PG45 also contained distinct restriction modification (RM) systems, which include three distinct types (I, II and III). The type I RM system usually includes a multifunctional enzyme that comprises three subunits encoded by three closely linked genes, *hsd*R, *hsd*M, and *hsd*S, which have both restriction and modification activities. A 13.0 kb fragment (276,441 nt to 289,403 nt) in HB0801 contained a complete type I RM system that was designated as *hsd*-2 (shown in purple in Figure 3A-2) and a recombinase. This fragment seemed to be completely different at the nucleotide level from the 10.2 kb fragment at the same position (703,157 nt to 713,368 nt) of the PG45 genome, which encoded a complete *hsd*-2 and an integrase. In addition, there was another fragment encoding a complete *hsd*-1 (Figure 3A-1) and a recombinase in HB0801 at separate positions (755,964 nt to 766,464 nt). Of the *hsd*-1 genes, *hsd*M and *hsd*R showed a high homology (93% and 99%) to those in the PG45 genome at same position (272,909 nt to 282,015 nt). However, this RM-specific subunit gene *hsd*S, which determines bacterial restriction and methylation specificity, varies between the two strains and experiences DNA rearrangement. The rearrangement in *hsd*S is believed to respond to environmental change and might be mediated by the adjacent integrase. There were two other fragments related to the RM system that varied between the HB0801 and PG45 genomes. In HB0801, one fragment (619,537 nt to 624,534 nt) contained a methyltransferase that was different from the corresponding fragment (412,382 nt to 415926 nt) in PG45, which contained a type II RM system. The other fragment (867,161 nt to 871451 nt) contained a type III RM system methylase pseudogene which was resulted from one frameshift mutation. This type III RM system methylase pseudogene varied from the corresponding fragment (866,349 nt to 871,012 nt), which also contained a degenerate type III RM system in PG45. Distinct RM systems of HB0801 and PG45 might imply their different abilities to adapt to the environment.

Another significantly varied region between the strains was marked with blue ovals in Figure 3A-2 and referred to the *vsp* cluster. HB0801 which has less and different *vsp* ORFs compared to PG45. Further analysis related to *vsp* cluster had been mentioned before.

In addition to the Vsp family, some putative lipoproteins (marked with green dots) and transmembrane proteins (marked with red dots) were shown to vary between HB0801 and PG45 (Figure 3A). A special fragment (595,710 nt to 613,545 nt) in HB0801, encoding five putative transmembrane proteins (*Mbov*_0510, 0514, 0516, 0517 and 0519) and two putative lipoproteins (*Mbov*_0515 and 0518), showed only 76% identities to the corresponding PG45 fragment (421,848 nt to 439,718 nt) by BLASTn analysis. The difference in the lipoproteins and transmembrane proteins may have resulted from varied environment pressures and may be important in pathogenesis and immune adaptation.

### Comparison between HB0801 and Hubei-1

The HB0801 genome was 43581 bp greater than Hubei-1. Based on the published Hubei-1 sequence (GenBank Accession no: CP002513), the size difference mainly involved two unique deletion fragments and nine insertion fragments in HB0801 (Figure 3B). One deletion fragment contained an IS*Mbov*1 (MMB_0191) and a hypothetical protein (MMB_0190), while another encoded an N-terminal truncated type III RM system methylase (MMB_0159).

Among the nine HB0801 insertion fragments, six were stand-alone mobile IS elements, including five IS*Mbov*3 copies and one copy of IS*Mbov*1.

Amazingly, the *vsp* gene cluster, which existed in both PG45 and HB0801, was completely deleted in Hubei-1, although the vicinal gene encoding an integrase-recombinase (xerC) remained. The *vsp* cluster was previously reported as a characteristic region in *M. bovis* [33].

In addition, there were three large insertion fragments in HB0801, which were 11.5 kb, 10.7 kb and 6.6 kb, respectively. The 11.5 kb insertion fragment (208,970 nt to 220,444 nt) contained two IS elements (IS*Mbov*1, IS*Mbov*2) and three putative lipoproteins (*Mbov*_0182, 0186 and 0188). The 10.7 kb (527,734 nt to 538,505 nt) insertion fragment consisted of four IS elements (IS*Mbov*1, IS*Mbov*2 and two copies of IS*Mbov*3) and three putative lipoproteins (*Mbov*_0458, *Mbov*_0461 and *Mbov*_0462) and the 6.6 kb insertion fragment (712,168 nt to 718,846 nt) encompassed a 16S rRNA, 23S rRNA and an IS*Mbov*4 psuedogene. Each insertion fragment contained mobile IS elements, which were suspected of mediating the fragment transfers. In contrast to the two 16S-23S rRNA operons in HB0801and PG45, there was only one in Hubei-1. However, two 5S rRNAs were found at other positions in the Hubei-1 genome. These findings indicated that Hubei-1 may have lost one 16S-23S rRNA operon as a result of internal IS*Mbov*4-mediated transfer.

Furthermore, two putative lipoproteins (*Mbov*_0339 and *Mbov*_0656) were found to possess intragenic insertion fragments in HB0801 compared to Hubei-1. The former which contained repeat sequences was inserted by two different repeat sequences of 39 bp at the repeat region. It showed a high homology to *vpma*Y1 in Hubei-1 and was designated as *vsp*Y1 in HB0801. The latter also contained repeat sequences and the 51 bp insertion fragment occurred at either repeat region. The repeat regions could lead to polymerase slippage during replication and result in the high frequency phase variation.

Meanwhile, we analyzed single nucleotide polymorphism (SNP) loci between the HB0801 and Hubei-1 genomes (Table S6). A total of 122 SNP loci were found. Among them, 57% (69/122) were present in IS elements (25 in IS*Mbov*1, 31 in IS*Mbov*7, 6 in IS*Mbov*2, 6 in IS*Mbov*3 and 1 in IS*Mbov*5). This suggested that the gene mutations were more likely to occur in IS elements during evolution. In addition, there were 18 SNPs present in six genes (*Mbov*_0339, *Mbov*_0473, *Mbov*_0477, *Mbov*_0518, *Mbov*_0730, *Mbov*_0732 and *Mbov*_0856), which encoded putative LPs. Of these, 10 were present in one gene (*Mbov*_0339) encoding for the VpmaY1-like variable surface lipoprotein (VspY1) and located near the two inserted fragments. Furthermore, there were five SNPs occurring in four different putative transmembrane proteins. The lipoproteins such as VspY1 and membrane proteins are thought to be potential virulence factors. Therefore, the corresponding SNPs may affect *M. bovis* pathogenicity. Moreover, there were six SNPs present in genes encoding transporters for nutrients, including glycerol (*Mbov*_0271), multiple sugars (*Mbov*_0581), oligopeptides (*Mbov*_0034 and *Mbov*_0037), and chromate (*Mbov*_0762), and a ABC transporter for drug resistance (*Mbov*_680). It is interesting that there also was the phosphoglycerate mutase gene (*Mbov*_0776), which functions in glycerol metabolism possessing one SNP locus. There were also two lipoate-protein ligase A (*lplA*) genes (*Mbov*_0009 and 0010) found to contain one SNP each. LplA is an essential enzyme to ligate free lipoate to target proteins and was found to affect the pathogenic virulence [34]. Almost all SNPs present in transporters resulted in missense mutations, except the SNP in the chromate transporter. Since glycerol is considered the main carbon and energy source of *M. bovis* [6], the SNPs present in genes responsible for the transport and metabolism of glycerol may affect *M. bovis* growth and pathogenicity. In addition, the SNPs that occur in drug resistance transporters may affect *M. bovis* drug resistance. However, this needs to be further analyzed.

### Comparative analysis between *M. bovis* and *M. agalactiae* strains

*M. agalactiae* is phylogenetically closest to *M. bovis* and about 89.6% of *M. bovis* genes showed homology to *M. agalactiae*. However, they tended to infect different ruminant hosts. We made a comparative analysis using the completed genome sequences of *M bovis* strains (PG45 and Hubei-1) and *M.agalactiae* strains (PG2 and 5632) to explore the genetic diversity between the two species. The results showed that the diversity involved genes encoding variable surface lipoproteins, putative lipoproteins, membrane proteins and some function-unknown hypothetical proteins (Table S7). Lipoproteins have been shown to have virulence-associated functions, such as colonization and invasion of hosts, and evasion of host immunodulation [35]. Similarly, membrane proteins are also known to play an important role in the interaction between pathogens and hosts. Therefore, the genetic diversity of lipoproteins and membrane proteins may help us to better understand the mechanism of host specificity of these pathogens.

The *M. bovis vsp* cluster was also compared to the corresponding *vpma* cluster in *M. agalactiae*. There is one *vpma* cluster in *M. agalactiae* PG2, but two clusters in *M. agalactiae* 5632. The sequences of *vpma* clusters in different strains of *M. agalactiae* varied and showed low homology to the *M. bovis vsp* clusters. However, it was common for both *vpma* and *vsp* genes to share highly conserved 5′ untranslated regions and short N-terminal sequences [36] and the gene clusters were close to a site-specific *xer* recombinase.

### Comparative analysis with other *Mycoplasmas*

**Figure 4:**
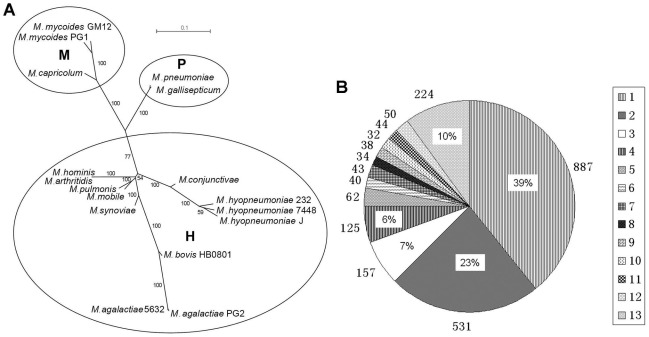
Phylogenetic Analysis and Orthologous Gene Detection. (A) Phylogenetic relationships of consensus sequences of 17 *Mycoplasma* strains with complete genomic sequences. The phylogenetic groups, mycoides, pneumoniae and hominis, are indicated by M, P and H, respectively. (B) Frequency of orthologous genes within the 13 genomes of different *Mycoplasma* species included in this analysis are listed in Table 1 (marked with *). The figures 1 to 13 on the right panel represent the number of genomes where the common orthologous genes were found. The rates in different parts of the circle represent the frequency of genes present in a single genome or shared by different genomes. The figures surrounding the circle represent orthologous gene numbers in individual parts. The 39% of genes including 887 present in a single genome represent lineage specific genes, while 10% of the genes including 224 were found in all 13 genomes, which represent the *Mycoplasma* core genome.

The orthologs of HB0801 and 16 other sequenced *Mycoplasmas* were searched using OrthoPC and a table describing gene content of various Mycoplasma genomes was developed (Table S1). Furthermore, a phylogenic tree of each ortholog and a super-tree was constructed (Figure 4A). The analyzed *Mycoplasmas* were divided into three clusters represented by mycoides (M), pneumoniae (P) and hominis (H). In cluster H, about 89.6% of *M. bovis* genes showed homology to *M. agalactiae* [35]. However, *M. mycoides* and *M. bovis* were situated in different clusters.

Furthermore, we selected 13 different species of *Mycoplasmas* by excluding the repeated genomes of strains for each species to estimate the genes of a *Mycoplasma* pan-genome. A surprisingly low ratio (39%) of genes in the pan-genome was discovered to exist in only one lineage, which suggested a remarkable degree of horizontal gene transfer in shaping *Mycoplasmas* genomes (Figure 4B). The genes shared by two genomes and three genomes occupy 23% and 7%, respectively. There were only 224 core genes present in all 13 genomes, which represented 10% of the total orthologous genes. The core *Mycoplasmas* genes encode proteins involved in essential cellular functions, such as ribosomal structure and biogenesis, DNA replication, transcription and translation, protein synthesis, energy production and conversion, and metabolism of nucleotides, carbohydrates, amino acids and inorganic ions (Table S2).

The lineage-specific genes present in all the 3 *M. bovis* strains were analyzed and the results are shown in Table S8. A total of 18 orthologs were predicted to be lineage-specific genes, which may be related to particular *M. bovis* characteristics. Among these, 7 genes encoded putative transmembrane proteins or putative lipoproteins, 2 encoded variable surface lipoproteins and 9 encoded hypothetical proteins. These specific membrane proteins and lipoproteins may be responsible for the special interaction between *M. bovis* and hosts.

### Horizontal gene transfer (HGT)

The HGT detection indicated that 27 orthologs might have undergone recombination between *M. bovis* HB0801 and other *Mycoplasma* species. Furthermore, 28 orthologs specific to *M. bovis* might have transferred between *M. bovis* with bacteria not analyzed in this paper (Table S3). In addition, we detected 107 HGT orthologs shared by *M. bovis* and *M. agalactiae* with other *Mycoplasma* species, other than *M. synoviae* (Table S4). We suppose that there might be a common ancestor shared by *M. bovis* and *M. agalactiae*, as the putative HGT genes may have been transferred between their ancestor and other *Mycoplasmas* species, and then passed along by both strains.

Interestingly, among the above HGT orthologs, 76 might have been putatively transferred between *M. bovis* and the phylogenetically remote mycoides cluster, including *M. mycoides subsp. mycoides* SC, *M. mycoides subsp. capristr* and *M. capricolum subsp. Capricolum*, as may be the case in *M. agalactiae* PG2 (Table S5) [37], in which 18 were putatively transferred only between *M. bovis* and the mycoides cluster (Table S3), while 58 may have been transferred between the mycoides cluster and both *M. bovis* and *M. agalactiae* (Table S4). Among these 18 orthologs, most genes function in replication, recombination and repair activities, such as transposases IS*Mbov*2, IS*Mbov*3 and IS*Mbov*7, DNA-methyltransferase (*Mbov*_0202, *Mbov*_0727, *Mbov*_0755 and *Mbov*_0708), DNA adenine methylase (*Mbov*_0709), and DNA and RNA helicases (*Mbov*_0647). In addition, there were four orthologs (*Mbov* _0253, *Mbov* _0340, *Mbov* _0380, and *Mbov* _0381) encoding putative transmembrane proteins and three encoding putative lipoproteins (*Mbov*_0049, *Mbov*_0473, *Mbov*_0350 and *Mbov*_0505) that may be responsible for immune adaptation.

Of the 58 orthologs previously mentioned, there were genes that played important roles in transportation or utilization of environmental nutrients such as oligopeptides, phosphonates, lactates and glycerol. They include an oligopeptide ABC transporter (OppFDCBA, *Mbov*_0033-0037), a phosphonate ABC transporter locus (phnDCE, *Mbov*_0306-0308), a D-lactate dehydrogenase (*Mbov*_0160), two clusters of glycerol ABC transporters (*Mbov*_0271-0273, *Mbov*_0740-0742) and a glycerol kinase (glpK, *Mbov*_0325). In addition, there was a set of genes reportedly involved in energy production, synthesis of amino acids or coenzymes including ATPase (atpDA, *Mbov*_0508-0509), and some ligases, including aspartate–ammonia ligase (asnA, *Mbov*_0071) and lipoate-protein ligase A (*lplA*, *Mbov*_0009 and *Mbov*_0010). Furthermore, some transferred genes responsible for environmental adaption were involved, such as the exodeoxyribonuclease V (recD, *Mbov*_0238), which was associated with recombination and repair, a peptide methionine sulfoxide reductase (MsrAB, *Mbov*_0488), endopeptidase O (pepO, *Mbov*_0428), periplasmic proteases with unknown function (*Mbov*_0658) and an ABC-2 type transporter ATP-binding protein (*Mbov*_0535), which was related to the multidrug transporter system. Further, what interested us were some virulence-associated genes, the NADH oxidase (hcaD, *Mbov*_0286) which might cause oxidative damage to the cellular membrane and two putative abortive infection proteins AbiGII (*Mbov*_0799-0800). There were also some putative lipoproteins and some transmembrane proteins, which were membrane-associated might play a role in pathogenesis.

### Sequence confirmation for inversion and vsp cluster

**Figure 5:**
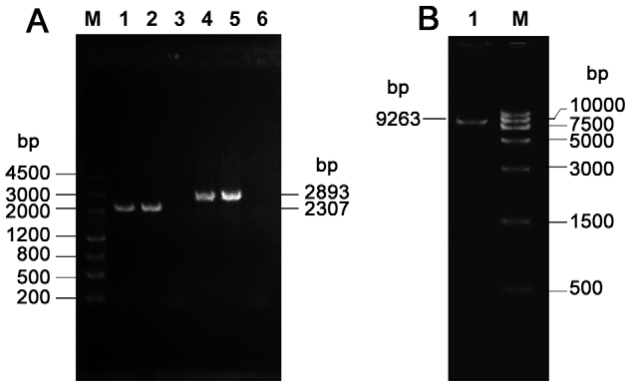
PCR Confirmation of Inversion and *vsp* Gene Cluster. (A) PCR amplification specific to the upstream and downstream connection sites of the *M. bovis* HB0801 inversion region. Lanes 1–3 represent PCR products using the primer Inv-1 specific to upstream connection region of the inversion. Lanes 4–6 represent PCR products using primer Inv-2 specific to the downstream connection region of the inversion. Lanes 1 and 4 used the HB0801 genome with pneumonia origin as the template, Lanes 2 and 5 used the HB1007 genome with mastitis origin as the template, and Lanes 3 and 6 represent PCR products using the *M. bovis* ATCC® 25523™/PG45 genome with mastitis origin as the template. (B) PCR product of the *vsp* region in the HB0801 genome.

PCR analysis specific to the adjoining regions at both ends of the inversion was performed and the products were sequenced. As expected, two fragments of 2307 bp and 2893 bp were obtained upstream and downstream of the inversion, respectively. After sequencing, both fragments showed 99.9% identities to the corresponding sequences of the HB0801 genome. These results confirmed an inversion in HB0801, a strain isolated from the lung of a pneumonic beef cattle (Figure 5A). To determine whether this inversion was specific to a pneumonia-causing *M. bovis* strain, we subsequently investigated strain HB1007 from milk of a dairy cow in Hubei-like *M. bovis* PG45 by PCR and sequencing. The results demonstrated that the inversion also occurred in HB1007 (Figure 5A).

The much shorter region for the *vsp* gene cluster in HB0801 was also verified by PCR. The PCR specific to the two flanking regions produced a fragment of 9263 bp and the size was consistent with the HB0801 sequencing results, indicating that the *vsp* region was sequenced correctly (Figure 5B).

### Virulence of *M. bovis* PG45 and HB0801 in cattle

The mock-infected control group did not have obvious clinical signs and their rectal temperatures fluctuated less than 0.5°C. In contrast, the two infected groups experienced fever between days 3 and 7 post-challenge with temperatures increasing slightly more than 1°C. In addition, the calves from both infected groups had a mild increase of thin nasal discharge.

The gross pathological lesions of the inner organs were scored according to severity. The total score of the control group and the PG45- and HB0801-infected groups were 3, 13 and 9, respectively. The infected groups had apparently more serious lesions than the control calves (Table 4).

**Table 4:** The Gross Pathological Assessment of Inner Organs.

	Groups		
	PG45	HB0801	Control
Calves(heads)	3	3	3
Lung serosal surface	6	3	0
Lung color	2	0	1
Pleural adhesion	3	1	1
*Pleural* effusion	2	4	1
Pericardial wall	0	1	0
Joint *effusion*	0	0	0
**Total score**	**13**	**9**	**3**

The pathological bronchial lesions were further scored by evaluating the lesions in sliced lung tissues. The total scores of the PG45 and HB0801 groups were 70 and 69, respectively, while that of control group was 36, which was significantly lower than those of PG45- (*P*<0.01) and HB0801- (*P*<0.01) infected groups (Table 5).

**Table 5:** The Assessment of Lung Lesions.

	Groups		
	PG45	HB0801	Control
Calves(heads)	3	3	3
Left apical	10	8	4
Left cardiac	8	4	4
Left diaphragmatic	8	9	6
Right apical	13	15	6
Right cardiac	8	10	2
Right diaphragmatic	12	13	5
Right accessory lobe	11	10	9
**Total score**	**70**	**69**	**36**

**Figure 6:**
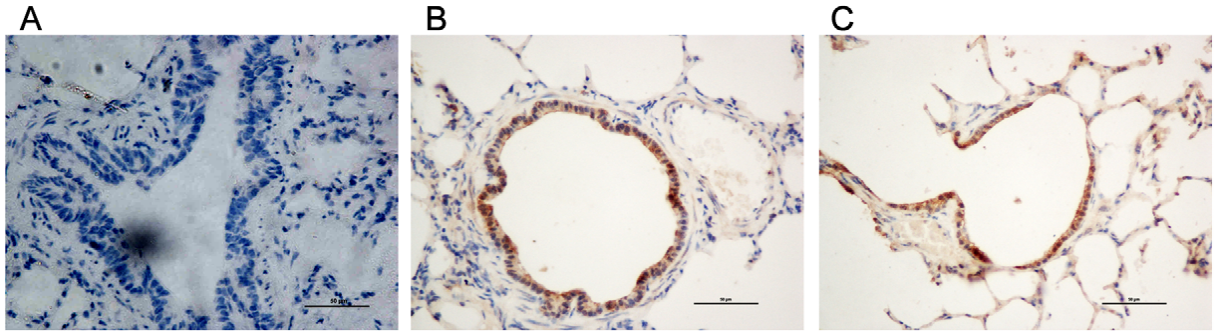
Immunohistochemical Staining of Lung Sections of Calves with *M.bovis* Infection. The lung tissues of calves from the negative control group (A), *M. bovis* HB0801- (B) and PG45-infected groups (C) were detected after immunohistochemical staining with monoclonal antibody to *M. bovis*. The positive cells were stained brown and the negative cells were counter-stained blue with hematoxylin. The positive cells were situated in bronchiole epithelia.

Next, we immunohistochemically detected *M. bovis* antigens in lung tissue and lymph nodes. Both tissue sections from the infected animals possessed many positive cells (stained brown). In lymph nodes, the positive cells were mainly located in the cytoplasm of macrophages (data not shown), while in lung tissue, positive cells were situated in the bronchiole epithelia (Figure 6). Quantitative analysis demonstrated that average IOD values of positive cell signals from lymph node sections for the PG45- and HB0801-infected groups, and controls were 22634.09, 25108.00 and 243.48, respectively. There was an apparent difference between both infection groups and the negative control (*P*<0.01), but no significant difference existed between the infected groups (*P* = 0.78). The results indicated that the two *M. bovis* strains had a similar capability to successfully invade and colonize these tissues.

## Discussion

Genomic plasticity can be expressed by gene acquisition and loss for an evolutionary advantage. This evolution follows a reductive mechanism that leads to the loss of non-essential genes, but not genes responsible for key functions within the hosts for competitive survival [38]. The evidence from this paper supports this view. Compared to *M. bovis* ATCC® 25523™/PG45, the genome size of *M. bovis* HB0801 in this study decreased by 11702 bp, which was mainly characterized by fewer *vsp* genes and ICE. Even between two Chinese strains HB0801 and Hubei-1, which were isolated during the same year and in the same province, we found a great divergence, including genome size, *vsp* gene family and 122 SNPs.

In addition, we found and confirmed with PCR a large inverted fragment (580 kb) in the Chinese strains compared to PG45. Since we demonstrated that this inversion existed in both pneumonia-causing strains (HB0801 and mastitis-causing strain HB1007) and the Chinese strain Hubei-1 isolated from the lesioned lung tissue [6], it was concluded that this inversion was not related to tissue segregation of *M. bovis*. As strain PG45 was isolated in America in the early 1960s, while HB0801 was identified in China in 2008, it is hypothesized that the interval of approximately 50 years and the geographical difference might be responsible for this inversion.

Furthermore, this high genomic plasticity is in agreement with the results of comparative genome analysis for 19 strains from 13 different *Mycoplasm*a species. From this analysis, we found that only 10% of the total orthologous genes were core genes, which indicated that most *Mycoplasm*a genes had been laterally transferred between inter- or intra-species. Consequently, *Mycoplasm*a species such as *M. bovis* have been actively evolving.

The difference between strains HB0801 and Hubei-1 may be partially related to their geographical locations because they were isolated from diseased cattle introduced from different regions of China to Hubei province. However, we could not exclude the limitation of sequencing techniques leading to loss of some genetic information such as the entire *vsp* gene cluster and some insertion fragments.

Although *M. bovis* was originally identified as early as 1961 [1], we actually know very little about its pathogenesis and virulence genes. We analyzed the implication of gene acquisition and loss on pathogenesis of different stains and explored mechanisms underlying these phenomena such as the potential contribution of mobile IS elements, RM systems, variation in *vsp* gene cluster, lipoproteins, transmembrane proteins, and ICEs. However, there is no confirmatory evidence to support our hypotheses.

For the first time, we connected genomic differences of *M. bovis* strains to their phenotypes by comparing PG45 and HB0801 virulence in cattle. We successfully showed that both strains were pathogenic to cattle by invading lung and lymph nodes, and causing lung lesions, pleural adhesion, and pleural effusion, which are common clinical signs of *M. bovis* infection. Furthermore, PG45 seemed more virulent than HB0801 in cattle, as determined by the large difference of gross pathogenic scores and small difference in lung lesion scores. However, there was no statistically significant difference (*P*>0.05). Theoretically, two possibilities may exist in this situation. First, the disadvantages of our animal model might underestimate this difference. The number of animals was too few (only three) for statistical analysis. In addition, the calves were purchased from a local market may not be very sensitive to the challenge of this pathogen. We chose local calves because they were slow-growing, small and inexpensive. The exotic Simmental cattle were more susceptible to *M.bovis* infection and comprised the main population possessing *M. bovis* pneumonia in China [4]. Another possibility would be that although variance accumulation in the HB0801 genome generated a trend of virulence modification, it was not sufficient to significantly affect the virulence of the strain. Therefore, more work needs to be done to elucidate the underlying connection between genome plasticity and virulence.

## Supporting Information

Table S1 **Gene content for the 17 Selected** ***Mycoplasma*** **genomes.**
(XLS)

Table S2 **Core Genome Content of the 13 Analyzed** ***Mycoplasmas*** **(the sequence data refer to** **Table 1** marked with *).
(XLS)

Table S3 **Putative Transferred Orthologous Genes between others and** ***M. bovis*** **.**
(XLS)

Table S4 **Putative Transferred Orthologous Genes between other** ***Mycoplasmas*** **with both** ***M. bovis*** **and** ***M. agalactiae*** **.**
(XLS)

Table S5 **Putative Transferred Orthologous Genes between** ***mycoides*** **cluster and** ***M. bovis*** **or** ***M. agalactiae*** **.**
(XLS)

Table S6 **SNP locations in** ***M.bovis*** **strain HB0801 compared to Hubei-1.**
(XLS)

Table S7 **Different genes between** ***M.bovis*** **species and** ***M.agalactiae*** **species.**
(XLS)

Table S8 **Lineage-specific genes in** ***M.bovis*** **species.**
(XLS)
